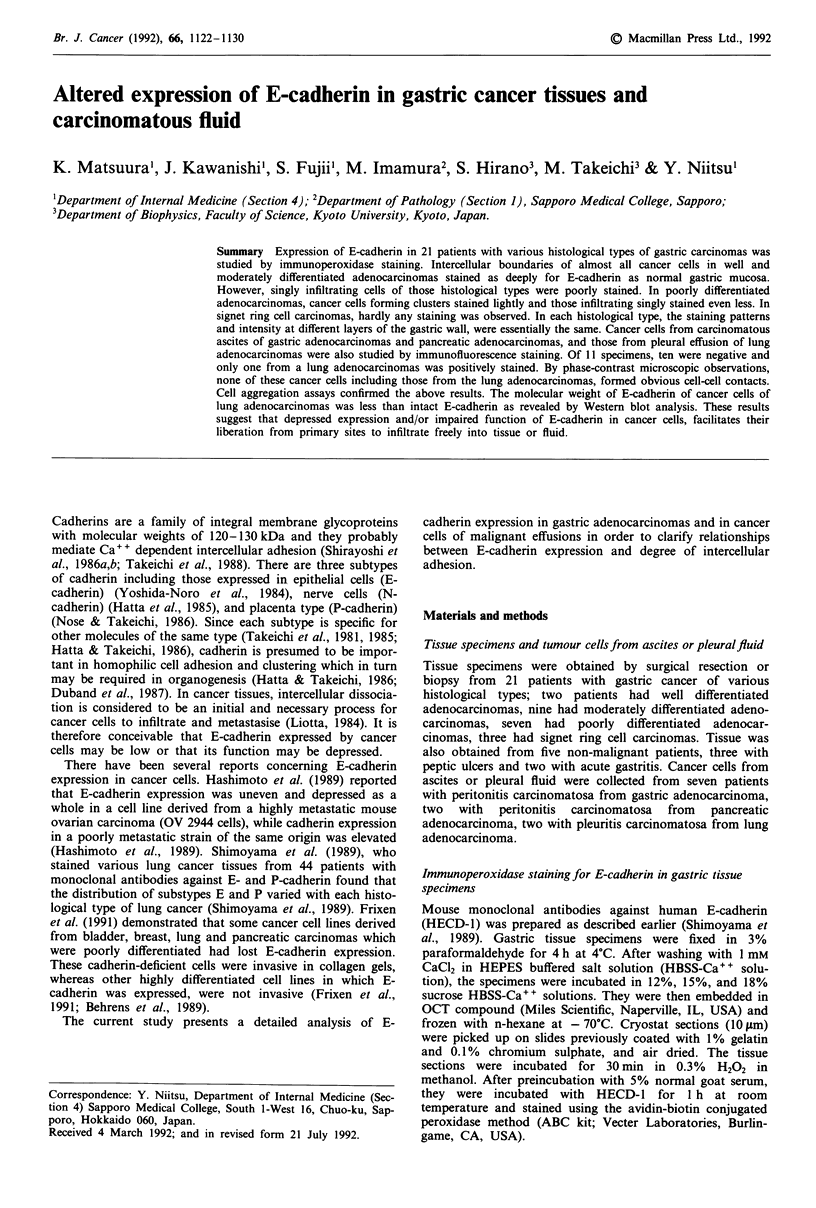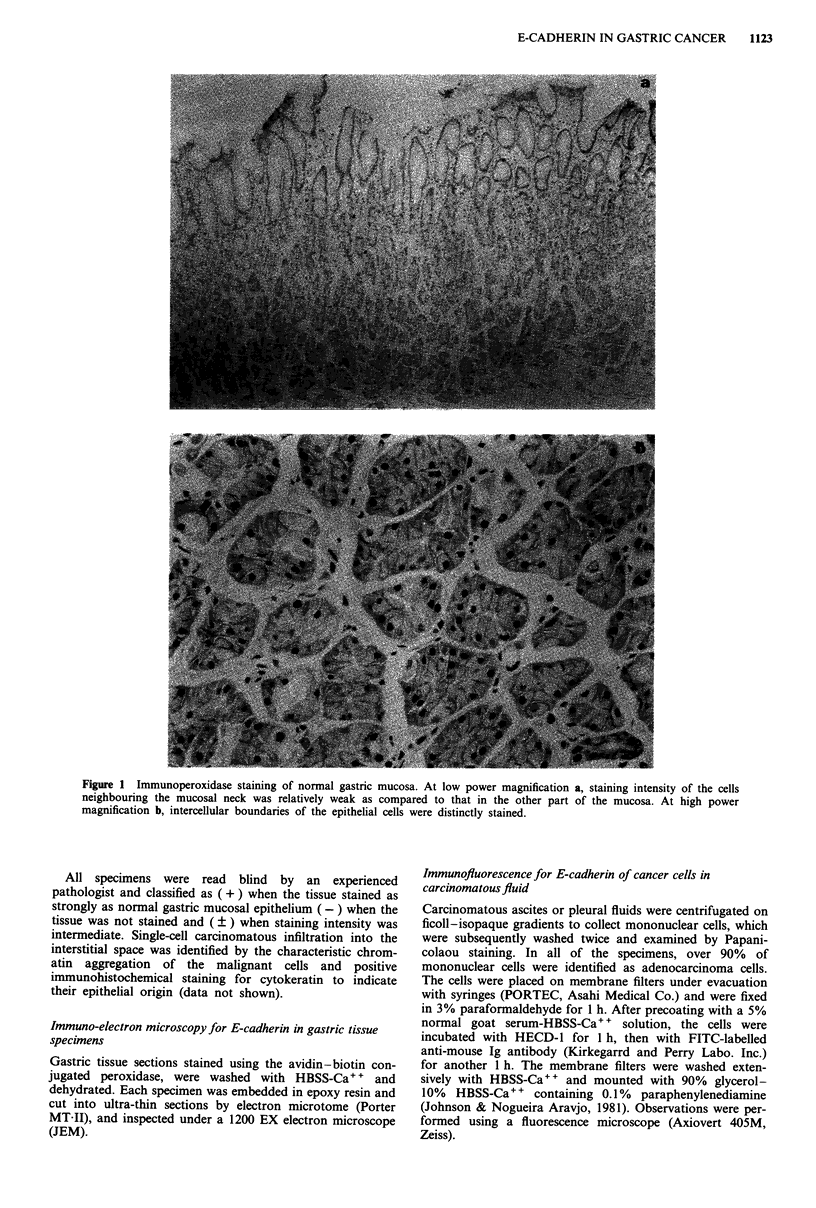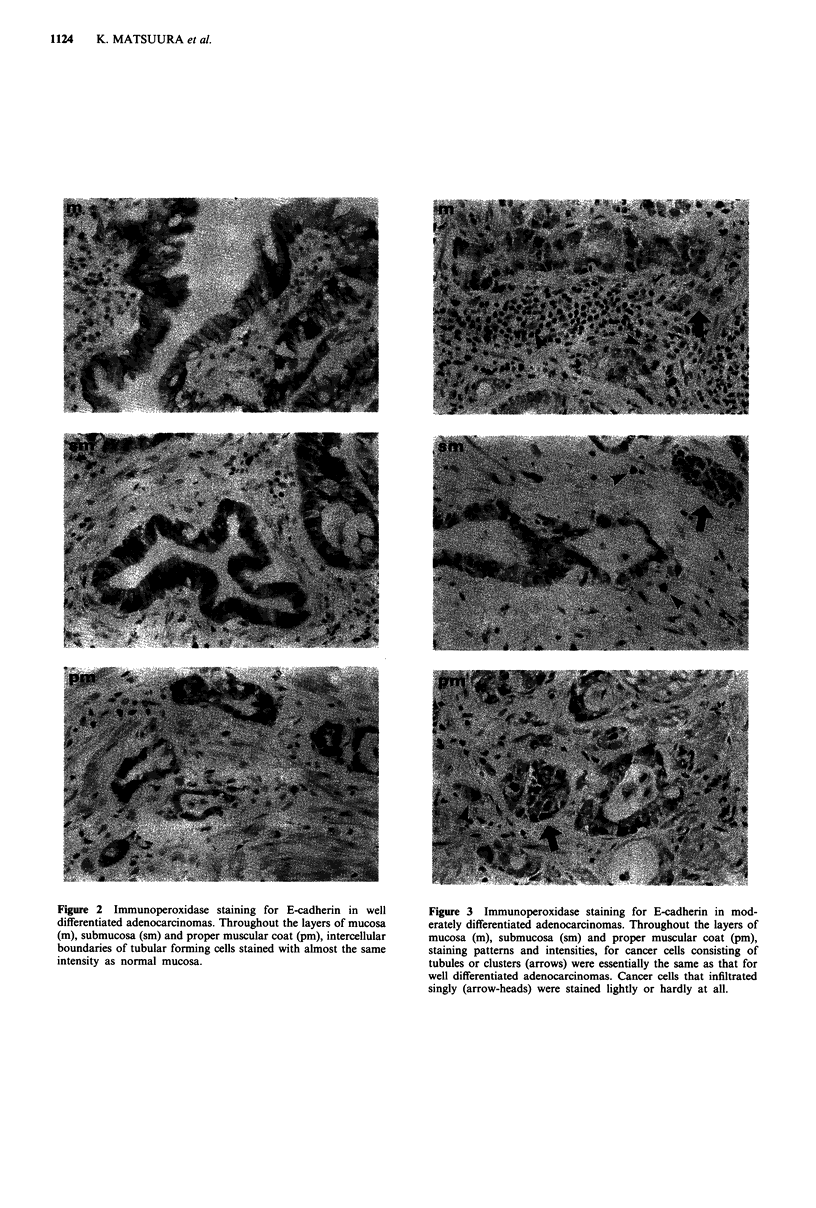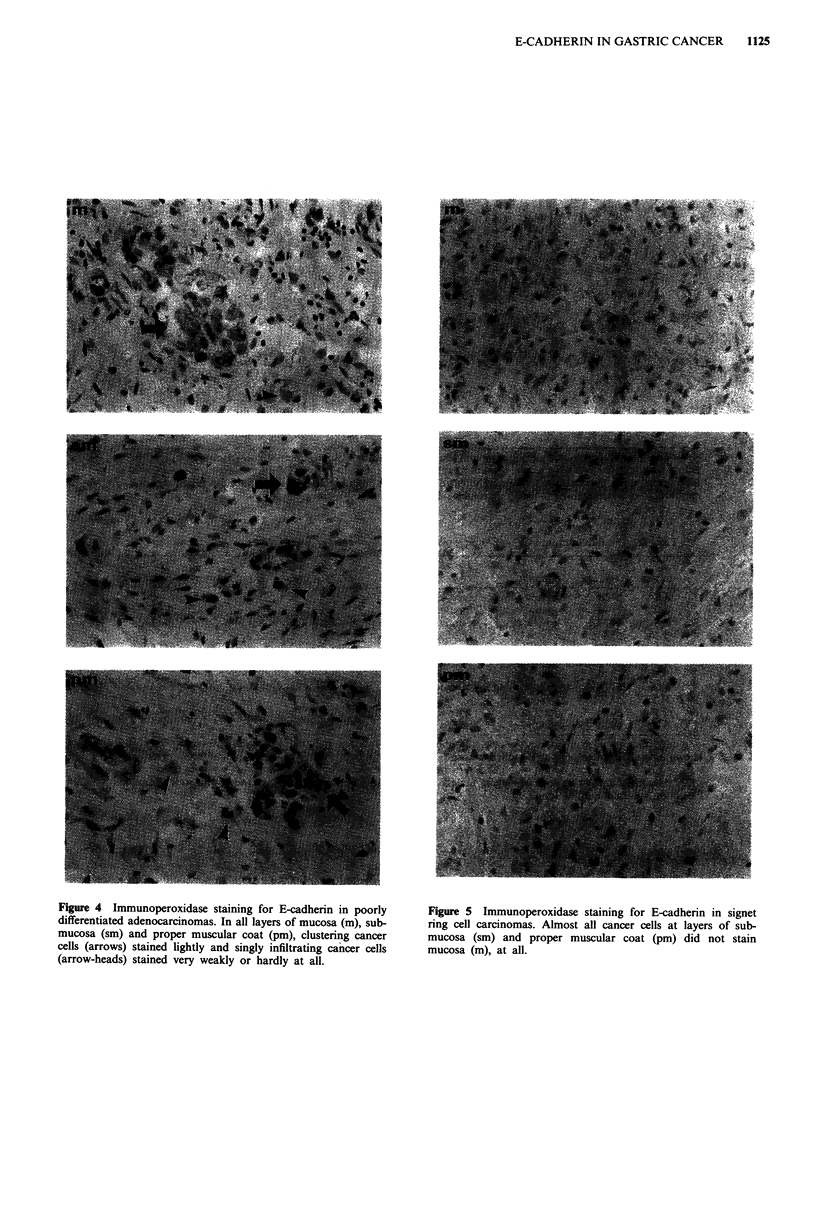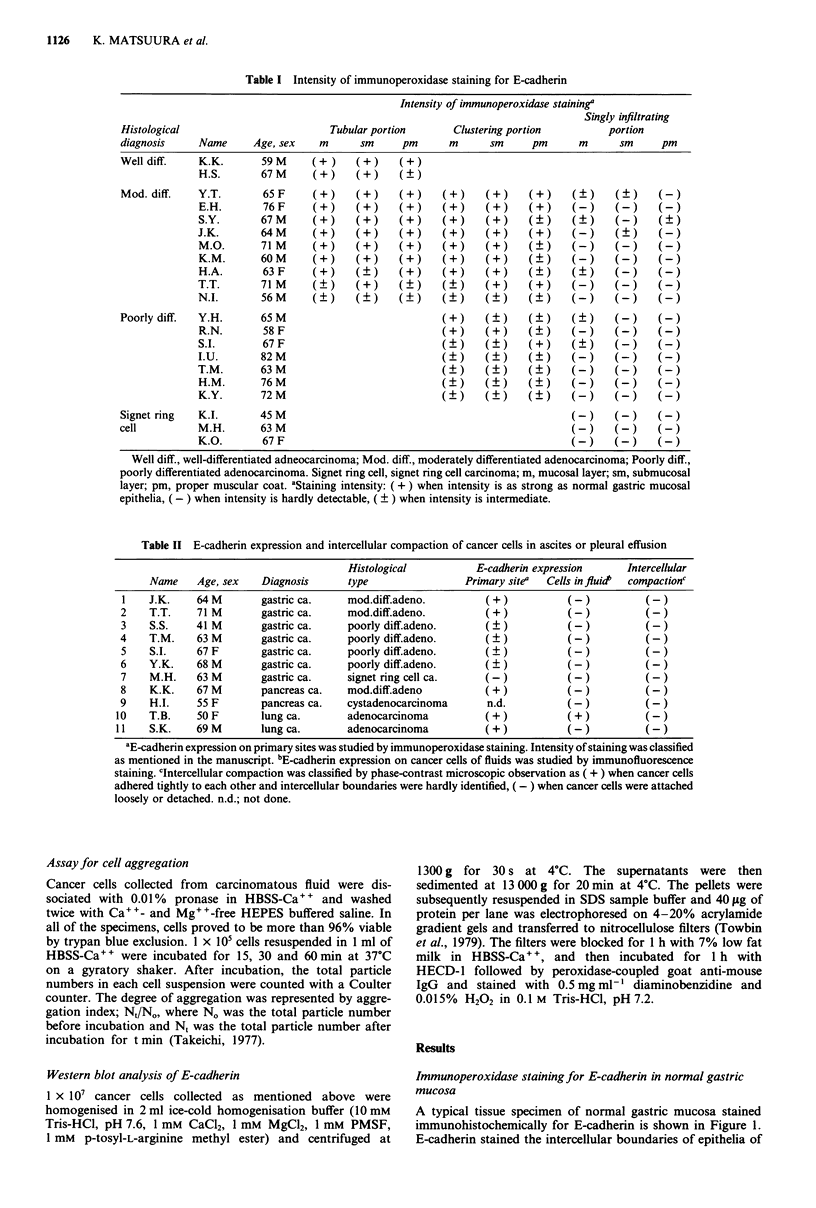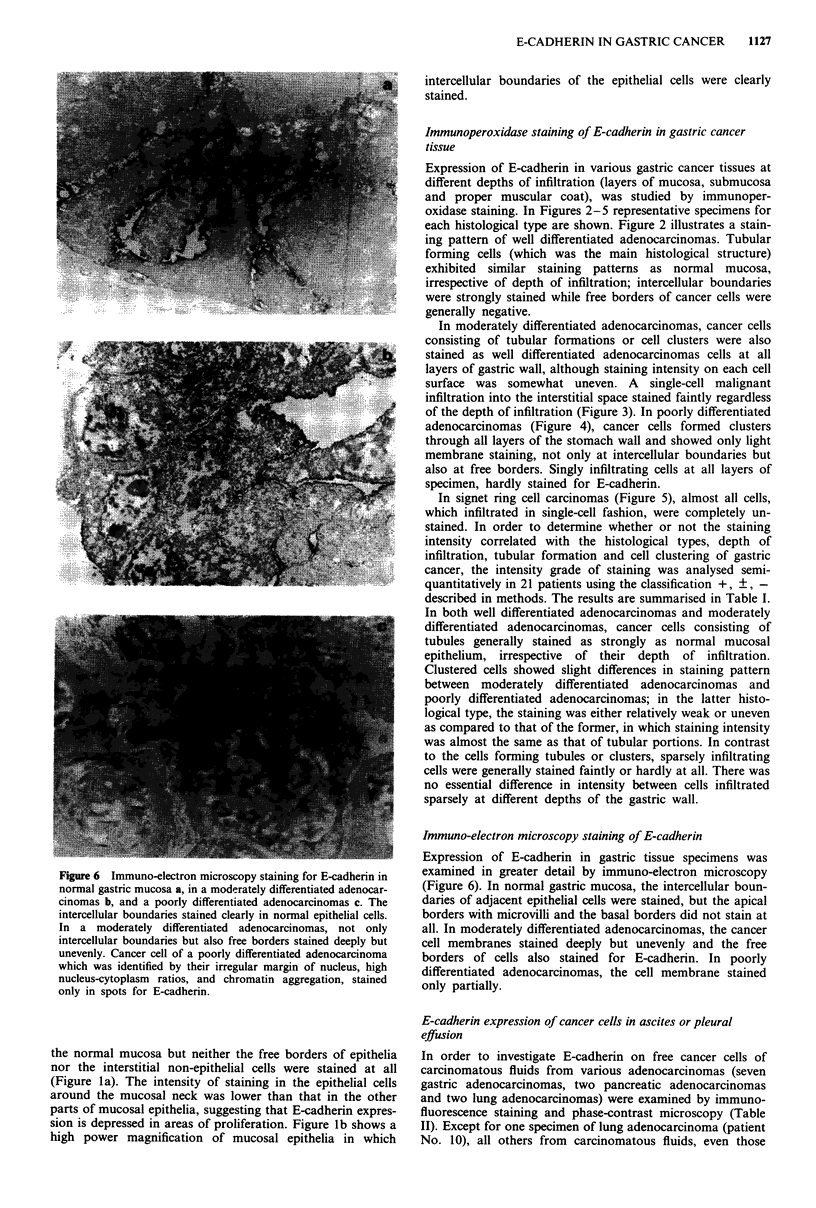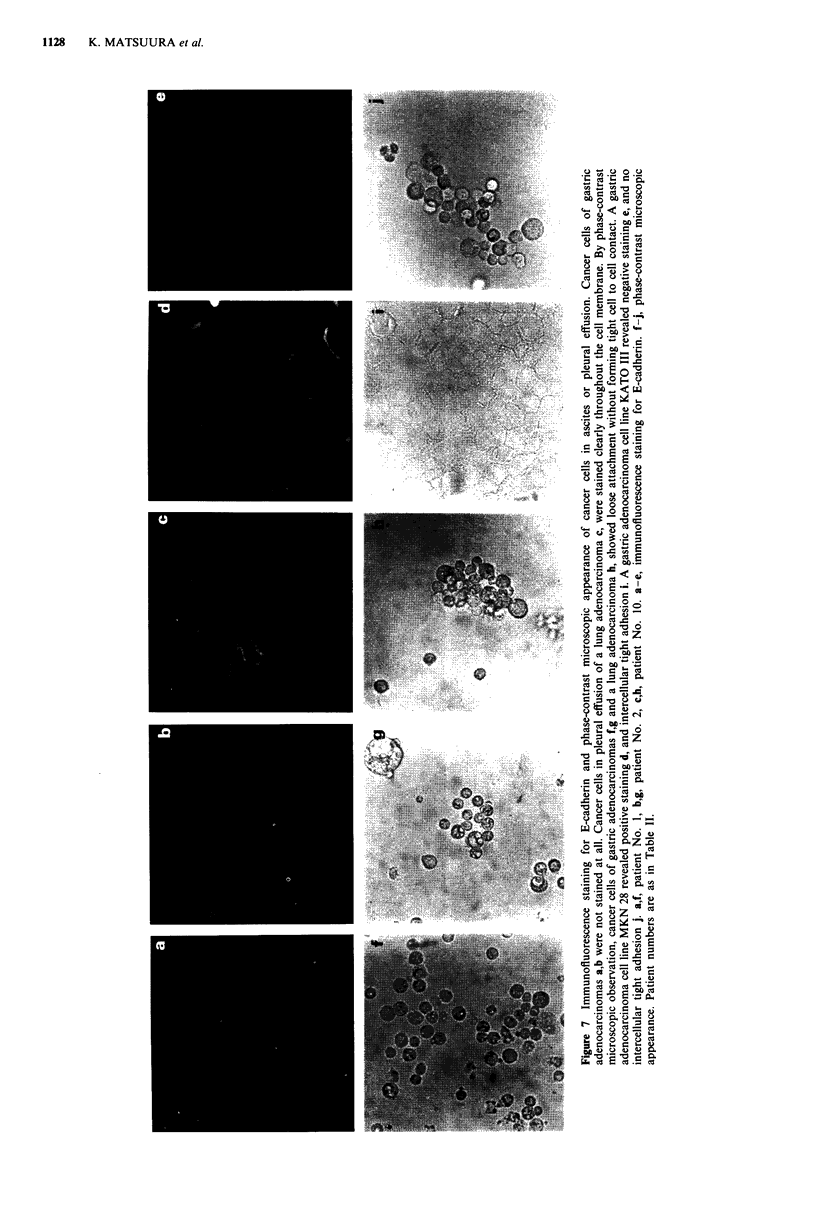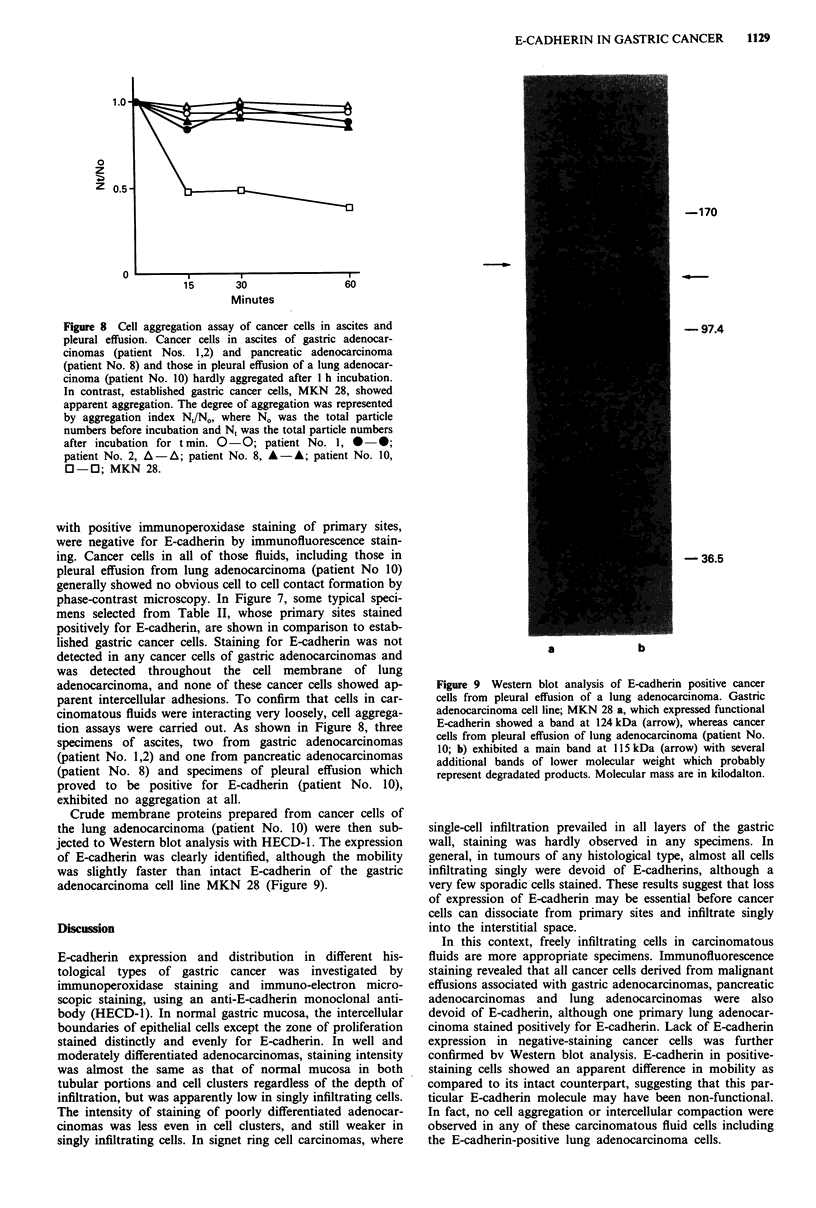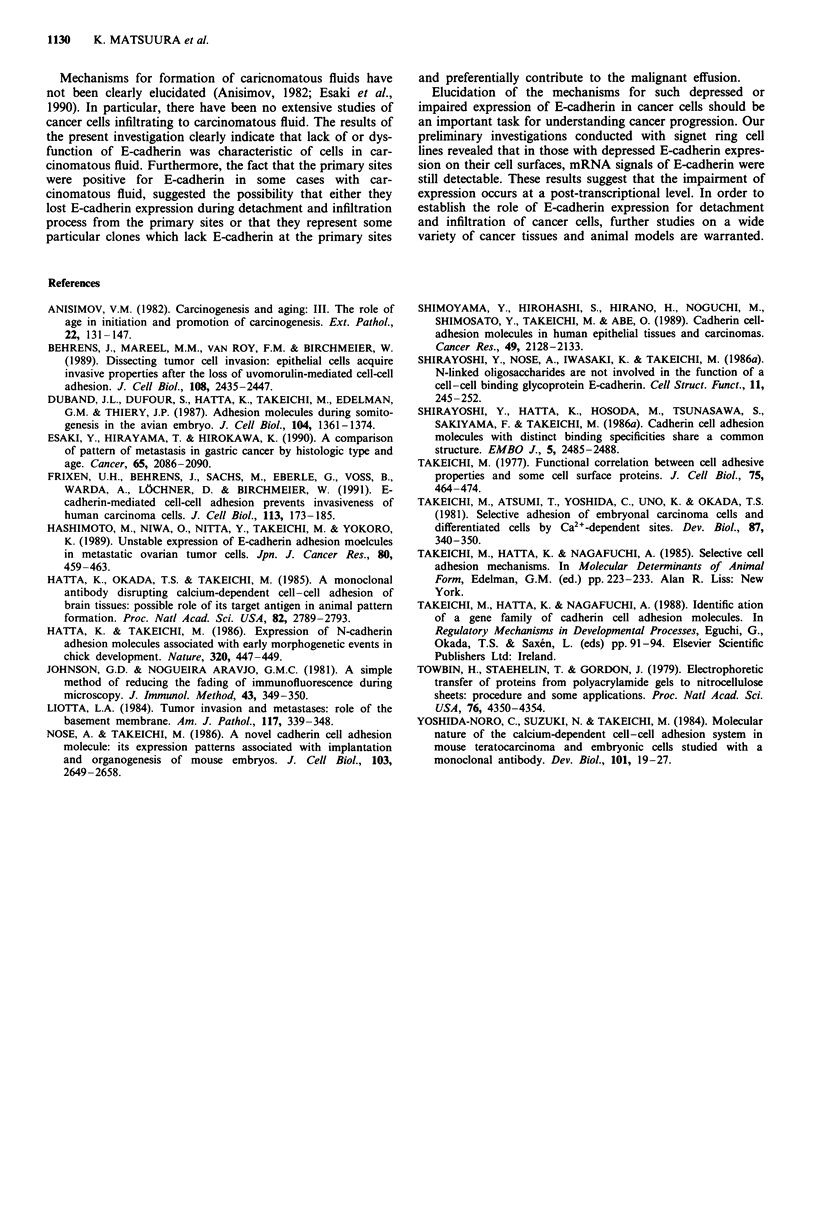# Altered expression of E-cadherin in gastric cancer tissues and carcinomatous fluid.

**DOI:** 10.1038/bjc.1992.421

**Published:** 1992-12

**Authors:** K. Matsuura, J. Kawanishi, S. Fujii, M. Imamura, S. Hirano, M. Takeichi, Y. Niitsu

**Affiliations:** Department of Internal Medicine (Section 4), Sapporo Medical College, Japan.

## Abstract

**Images:**


 © Macmillan Press Ltd., 1992

### Altered expression of E-cadherin in gastric cancer tissues and carcinomatous fluid

K. Matsuura[1], J. Kawanishi[1], S. Fujii[1], M. Imamura[2], S. Hirano[3], M. Takeichi[3] & Y. Niitsu[1]

[1]*Department of Internal Medicine (Section 4);* [2]*Department of Pathology (Section 1), Sapporo Medical College, Sapporo;* [3]*Department of Biophysics, Faculty of Science, Kyoto University, Kyoto, Japan.*

**Summary** Expression of E-cadherin in 21 patients with various histological types of gastric carcinomas was studied by immunoperoxidase staining. Intercellular boundaries of almost all cancer cells in well and moderately differentiated adenocarcinomas stained as deeply for E-cadherin as normal gastric mucosa. However, singly infiltrating cells of those histological types were poorly stained. In poorly differentiated adenocarcinomas, cancer cells forming clusters stained lightly and those infiltrating singly stained even less. In signet ring cell carcinomas, hardly any staining was observed. In each histological type, the staining patterns and intensity at different layers of the gastric wall, were essentially the same. Cancer cells from carcinomatous ascites of gastric adenocarcinomas and pancreatic adenocarcinomas, and those from pleural effusion of lung adenocarcinomas were also studied by immunofluorescence staining. Of 11 specimens, ten were negative and only one from a lung adenocarcinomas was positively stained. By phase-contrast microscopic observations, none of these cancer cells including those from the lung adenocarcinomas, formed obvious cell-cell contacts. Cell aggregation assays confirmed the above results. The molecular weight of E-cadherin of cancer cells of lung adenocarcinomas was less than intact E-cadherin as revealed by Western blot analysis. These results suggest that depressed expression and/or impaired function of E-cadherin in cancer cells, facilitates their liberation from primary sites to infiltrate freely into tissue or fluid.

Cadherins are a family of integral membrane glycoproteins with molecular weights of 120-130 kDa and they probably mediate $Ca^{++}$ dependent intercellular adhesion (Shirayoshi *et al.*, 1986*a*,*b*; Takeichi *et al.*, 1988). There are three subtypes of cadherin including those expressed in epithelial cells (E-cadherin) (Yoshida-Noro *et al.*, 1984), nerve cells (N-cadherin) (Hatta *et al.*, 1985), and placenta type (P-cadherin) (Nose & Takeichi, 1986). Since each subtype is specific for other molecules of the same type (Takeichi *et al.*, 1981, 1985; Hatta & Takeichi, 1986), cadherin is presumed to be important in homophilic cell adhesion and clustering which in turn may be required in organogenesis (Hatta & Takeichi, 1986; Duband *et al.*, 1987). In cancer tissues, intercellular dissociation is considered to be an initial and necessary process for cancer cells to infiltrate and metastasise (Liotta, 1984). It is therefore conceivable that E-cadherin expressed by cancer cells may be low or that its function may be depressed.

There have been several reports concerning E-cadherin expression in cancer cells. Hashimoto *et al.* (1989) reported that E-cadherin expression was uneven and depressed as a whole in a cell line derived from a highly metastatic mouse ovarian carcinoma (OV 2944 cells), while cadherin expression in a poorly metastatic strain of the same origin was elevated (Hashimoto *et al.*, 1989). Shimoyama *et al.* (1989), who stained various lung cancer tissues from 44 patients with monoclonal antibodies against E- and P-cadherin found that the distribution of substypes E and P varied with each histological type of lung cancer (Shimoyama *et al.*, 1989). Frixen *et al.* (1991) demonstrated that some cancer cell lines derived from bladder, breast, lung and pancreatic carcinomas which were poorly differentiated had lost E-cadherin expression. These cadherin-deficient cells were invasive in collagen gels, whereas other highly differentiated cell lines in which E-cadherin was expressed, were not invasive (Frixen *et al.*, 1991; Behrens *et al.*, 1989).

The current study presents a detailed analysis of E-cadherin expression in gastric adenocarcinomas and in cancer cells of malignant effusions in order to clarify relationships between E-cadherin expression and degree of intercellular adhesion.

Correspondence: Y. Niitsu, Department of Internal Medicine (Section 4) Sapporo Medical College, South 1-West 16, Chuo-ku, Sapporo, Hokkaido 060, Japan.
Received 4 March 1992; and in revised form 21 July 1992.

### Materials and methods

### *Tissue specimens and tumour cells from ascites or pleural fluid*

Tissue specimens were obtained by surgical resection or biopsy from 21 patients with gastric cancer of various histological types; two patients had well differentiated adenocarcinomas, nine had moderately differentiated adenocarcinomas, seven had poorly differentiated adenocarcinomas, three had signet ring cell carcinomas. Tissue was also obtained from five non-malignant patients, three with peptic ulcers and two with acute gastritis. Cancer cells from ascites or pleural fluid were collected from seven patients with peritonitis carcinomatosa from gastric adenocarcinoma, two with peritonitis carcinomatosa from pancreatic adenocarcinoma, two with pleuritis carcinomatosa from lung adenocarcinoma.

### *Immunoperoxidase staining for E-cadherin in gastric tissue specimens*

Mouse monoclonal antibodies against human E-cadherin (HECD-1) was prepared as described earlier (Shimoyama *et al.*, 1989). Gastric tissue specimens were fixed in 3% paraformaldehyde for 4 h at 4°C. After washing with 1 mM $CaCl_2$ in HEPES buffered salt solution (HBSS-$Ca^{++}$ solution), the specimens were incubated in 12%, 15%, and 18% sucrose HBSS-$Ca^{++}$ solutions. They were then embedded in OCT compound (Miles Scientific, Naperville, IL, USA) and frozen with n-hexane at $-70$°C. Cryostat sections (10 μm) were picked up on slides previously coated with 1% gelatin and 0.1% chromium sulphate, and air dried. The tissue sections were incubated for 30 min in 0.3% $H_2O_2$ in methanol. After preincubation with 5% normal goat serum, they were incubated with HECD-1 for 1 h at room temperature and stained using the avidin-biotin conjugated peroxidase method (ABC kit; Vecter Laboratories, Burlingame, CA, USA).

**Figure 1** Immunoperoxidase staining of normal gastric mucosa. At low power magnification **a**, staining intensity of the cells neighbouring the mucosal neck was relatively weak as compared to that in the other part of the mucosa. At high power magnification **b**, intercellular boundaries of the epithelial cells were distinctly stained.

All specimens were read blind by an experienced pathologist and classified as ( + ) when the tissue stained as strongly as normal gastric mucosal epithelium ( – ) when the tissue was not stained and ( ± ) when staining intensity was intermediate. Single-cell carcinomatous infiltration into the interstitial space was identified by the characteristic chromatin aggregation of the malignant cells and positive immunohistochemical staining for cytokeratin to indicate their epithelial origin (data not shown).

*Immuno-electron microscopy for E-cadherin in gastric tissue specimens*

Gastric tissue sections stained using the avidin–biotin conjugated peroxidase, were washed with HBSS-$Ca^{++}$ and dehydrated. Each specimen was embedded in epoxy resin and cut into ultra-thin sections by electron microtome (Porter MT·II), and inspected under a 1200 EX electron microscope (JEM).

*Immunofluorescence for E-cadherin of cancer cells in carcinomatous fluid*

Carcinomatous ascites or pleural fluids were centrifugated on ficoll–isopaque gradients to collect mononuclear cells, which were subsequently washed twice and examined by Papanicolaou staining. In all of the specimens, over 90% of mononuclear cells were identified as adenocarcinoma cells. The cells were placed on membrane filters under evacuation with syringes (PORTEC, Asahi Medical Co.) and were fixed in 3% paraformaldehyde for 1 h. After precoating with a 5% normal goat serum-HBSS-$Ca^{++}$ solution, the cells were incubated with HECD-1 for 1 h, then with FITC-labelled anti-mouse Ig antibody (Kirkegarrd and Perry Labo. Inc.) for another 1 h. The membrane filters were washed extensively with HBSS-$Ca^{++}$ and mounted with 90% glycerol–10% HBSS-$Ca^{++}$ containing 0.1% paraphenylenediamine (Johnson & Nogueira Aravjo, 1981). Observations were performed using a fluorescence microscope (Axiovert 405M, Zeiss).

**Figure 2** Immunoperoxidase staining for E-cadherin in well differentiated adenocarcinomas. Throughout the layers of mucosa (m), submucosa (sm) and proper muscular coat (pm), intercellular boundaries of tubular forming cells stained with almost the same intensity as normal mucosa.

**Figure 3** Immunoperoxidase staining for E-cadherin in moderately differentiated adenocarcinomas. Throughout the layers of mucosa (m), submucosa (sm) and proper muscular coat (pm), staining patterns and intensities, for cancer cells consisting of tubules or clusters (arrows) were essentially the same as that for well differentiated adenocarcinomas. Cancer cells that infiltrated singly (arrow-heads) were stained lightly or hardly at all.

**Figure 4** Immunoperoxidase staining for E-cadherin in poorly differentiated adenocarcinomas. In all layers of mucosa (m), submucosa (sm) and proper muscular coat (pm), clustering cancer cells (arrows) stained lightly and singly infiltrating cancer cells (arrow-heads) stained very weakly or hardly at all.

**Figure 5** Immunoperoxidase staining for E-cadherin in signet ring cell carcinomas. Almost all cancer cells at layers of submucosa (sm) and proper muscular coat (pm) did not stain mucosa (m), at all.

**Table I** Intensity of immunoperoxidase staining for E-cadherin

| | | | *Intensity of immunoperoxidase staining*[a] | | | | | | | | |
|---|---|---|---|---|---|---|---|---|---|---|---|
| *Histological diagnosis* | *Name* | *Age, sex* | *Tubular portion* *m* | *sm* | *pm* | *Clustering portion* *m* | *sm* | *pm* | *Singly infiltrating portion* *m* | *sm* | *pm* |
| Well diff. | K.K. | 59 M | (+) | (+) | (+) | | | | | | |
| | H.S. | 67 M | (+) | (+) | (±) | | | | | | |
| Mod. diff. | Y.T. | 65 F | (+) | (+) | (+) | (+) | (+) | (+) | (±) | (±) | (−) |
| | E.H. | 76 F | (+) | (+) | (+) | (+) | (+) | (+) | (−) | (−) | (−) |
| | S.Y. | 67 M | (+) | (+) | (+) | (+) | (+) | (±) | (±) | (−) | (±) |
| | J.K. | 64 M | (+) | (+) | (+) | (+) | (+) | (+) | (−) | (±) | (−) |
| | M.O. | 71 M | (+) | (+) | (+) | (+) | (+) | (±) | (−) | (−) | (−) |
| | K.M. | 60 M | (+) | (+) | (+) | (+) | (+) | (±) | (−) | (−) | (−) |
| | H.A. | 63 F | (+) | (±) | (+) | (+) | (+) | (±) | (±) | (−) | (−) |
| | T.T. | 71 M | (±) | (+) | (±) | (±) | (+) | (+) | (−) | (−) | (−) |
| | N.I. | 56 M | (±) | (±) | (±) | (±) | (±) | (±) | (−) | (−) | (−) |
| Poorly diff. | Y.H. | 65 M | | | | (+) | (±) | (±) | (±) | (−) | (−) |
| | R.N. | 58 F | | | | (+) | (+) | (±) | (−) | (−) | (−) |
| | S.I. | 67 F | | | | (±) | (±) | (+) | (±) | (−) | (−) |
| | I.U. | 82 M | | | | (±) | (±) | (±) | (−) | (−) | (−) |
| | T.M. | 63 M | | | | (±) | (±) | (±) | (−) | (−) | (−) |
| | H.M. | 76 M | | | | (±) | (±) | (±) | (−) | (−) | (−) |
| | K.Y. | 72 M | | | | (±) | (±) | (±) | (−) | (−) | (−) |
| Signet ring cell | K.I. | 45 M | | | | | | | (−) | (−) | (−) |
| | M.H. | 63 M | | | | | | | (−) | (−) | (−) |
| | K.O. | 67 F | | | | | | | (−) | (−) | (−) |

Well diff., well-differentiated adneocarcinoma; Mod. diff., moderately differentiated adenocarcinoma; Poorly diff., poorly differentiated adenocarcinoma. Signet ring cell, signet ring cell carcinoma; m, mucosal layer; sm, submucosal layer; pm, proper muscular coat. [a]Staining intensity: ( + ) when intensity is as strong as normal gastric mucosal epithelia, ( − ) when intensity is hardly detectable, ( ± ) when intensity is intermediate.

**Table II** E-cadherin expression and intercellular compaction of cancer cells in ascites or pleural effusion

| | *Name* | *Age, sex* | *Diagnosis* | *Histological type* | *E-cadherin expression* *Primary site*[a] | *Cells in fluid*[b] | *Intercellular compaction*[c] |
|---|---|---|---|---|---|---|---|
| 1 | J.K. | 64 M | gastric ca. | mod.diff.adeno. | (+) | (−) | (−) |
| 2 | T.T. | 71 M | gastric ca. | mod.diff.adeno. | (+) | (−) | (−) |
| 3 | S.S. | 41 M | gastric ca. | poorly diff.adeno. | (±) | (−) | (−) |
| 4 | T.M. | 63 M | gastric ca. | poorly diff.adeno. | (±) | (−) | (−) |
| 5 | S.I. | 67 F | gastric ca. | poorly diff.adeno. | (±) | (−) | (−) |
| 6 | Y.K. | 68 M | gastric ca. | poorly diff.adeno. | (±) | (−) | (−) |
| 7 | M.H. | 63 M | gastric ca. | signet ring cell ca. | (−) | (−) | (−) |
| 8 | K.K. | 67 M | pancreas ca. | mod.diff.adeno | (+) | (−) | (−) |
| 9 | H.I. | 55 F | pancreas ca. | cystadenocarcinoma | n.d. | (−) | (−) |
| 10 | T.B. | 50 F | lung ca. | adenocarcinoma | (+) | (+) | (−) |
| 11 | S.K. | 69 M | lung ca. | adenocarcinoma | (+) | (−) | (−) |

[a]E-cadherin expression on primary sites was studied by immunoperoxidase staining. Intensity of staining was classified as mentioned in the manuscript. [b]E-cadherin expression on cancer cells of fluids was studied by immunofluorescence staining. [c]Intercellular compaction was classified by phase-contrast microscopic observation as ( + ) when cancer cells adhered tightly to each other and intercellular boundaries were hardly identified, ( − ) when cancer cells were attached loosely or detached. n.d.; not done.

### *Assay for cell aggregation*

Cancer cells collected from carcinomatous fluid were dissociated with 0.01% pronase in HBSS-$Ca^{++}$ and washed twice with $Ca^{++}$- and $Mg^{++}$-free HEPES buffered saline. In all of the specimens, cells proved to be more than 96% viable by trypan blue exclusion. $1 \times 10^5$ cells resuspended in 1 ml of HBSS-$Ca^{++}$ were incubated for 15, 30 and 60 min at 37°C on a gyratory shaker. After incubation, the total particle numbers in each cell suspension were counted with a Coulter counter. The degree of aggregation was represented by aggregation index; $N_t/N_o$, where $N_o$ was the total particle number before incubation and $N_t$ was the total particle number after incubation for t min (Takeichi, 1977).

### *Western blot analysis of E-cadherin*

$1 \times 10^7$ cancer cells collected as mentioned above were homogenised in 2 ml ice-cold homogenisation buffer (10 mM Tris-HCl, pH 7.6, 1 mM $CaCl_2$, 1 mM $MgCl_2$, 1 mM PMSF, 1 mM p-tosyl-L-arginine methyl ester) and centrifuged at 1300 g for 30 s at 4°C. The supernatants were then sedimented at 13 000 g for 20 min at 4°C. The pellets were subsequently resuspended in SDS sample buffer and 40 μg of protein per lane was electrophoresed on 4–20% acrylamide gradient gels and transferred to nitrocellulose filters (Towbin *et al.*, 1979). The filters were blocked for 1 h with 7% low fat milk in HBSS-$Ca^{++}$, and then incubated for 1 h with HECD-1 followed by peroxidase-coupled goat anti-mouse IgG and stained with 0.5 mg $ml^{-1}$ diaminobenzidine and 0.015% $H_2O_2$ in 0.1 M Tris-HCl, pH 7.2.

### Results

### *Immunoperoxidase staining for E-cadherin in normal gastric mucosa*

A typical tissue specimen of normal gastric mucosa stained immunohistochemically for E-cadherin is shown in Figure 1. E-cadherin stained the intercellular boundaries of epithelia of

**Figure 6** Immuno-electron microscopy staining for E-cadherin in normal gastric mucosa **a**, in a moderately differentiated adenocarcinomas **b**, and a poorly differentiated adenocarcinomas **c**. The intercellular boundaries stained clearly in normal epithelial cells. In a moderately differentiated adenocarcinomas, not only intercellular boundaries but also free borders stained deeply but unevenly. Cancer cell of a poorly differentiated adenocarcinoma which was identified by their irregular margin of nucleus, high nucleus-cytoplasm ratios, and chromatin aggregation, stained only in spots for E-cadherin.

the normal mucosa but neither the free borders of epithelia nor the interstitial non-epithelial cells were stained at all (Figure 1a). The intensity of staining in the epithelial cells around the mucosal neck was lower than that in the other parts of mucosal epithelia, suggesting that E-cadherin expression is depressed in areas of proliferation. Figure 1b shows a high power magnification of mucosal epithelia in which intercellular boundaries of the epithelial cells were clearly stained.

*Immunoperoxidase staining of E-cadherin in gastric cancer tissue*

Expression of E-cadherin in various gastric cancer tissues at different depths of infiltration (layers of mucosa, submucosa and proper muscular coat), was studied by immunoperoxidase staining. In Figures 2–5 representative specimens for each histological type are shown. Figure 2 illustrates a staining pattern of well differentiated adenocarcinomas. Tubular forming cells (which was the main histological structure) exhibited similar staining patterns as normal mucosa, irrespective of depth of infiltration; intercellular boundaries were strongly stained while free borders of cancer cells were generally negative.

In moderately differentiated adenocarcinomas, cancer cells consisting of tubular formations or cell clusters were also stained as well differentiated adenocarcinomas cells at all layers of gastric wall, although staining intensity on each cell surface was somewhat uneven. A single-cell malignant infiltration into the interstitial space stained faintly regardless of the depth of infiltration (Figure 3). In poorly differentiated adenocarcinomas (Figure 4), cancer cells formed clusters through all layers of the stomach wall and showed only light membrane staining, not only at intercellular boundaries but also at free borders. Singly infiltrating cells at all layers of specimen, hardly stained for E-cadherin.

In signet ring cell carcinomas (Figure 5), almost all cells, which infiltrated in single-cell fashion, were completely unstained. In order to determine whether or not the staining intensity correlated with the histological types, depth of infiltration, tubular formation and cell clustering of gastric cancer, the intensity grade of staining was analysed semiquantitatively in 21 patients using the classification +, ±, − described in methods. The results are summarised in Table I. In both well differentiated adenocarcinomas and moderately differentiated adenocarcinomas, cancer cells consisting of tubules generally stained as strongly as normal mucosal epithelium, irrespective of their depth of infiltration. Clustered cells showed slight differences in staining pattern between moderately differentiated adenocarcinomas and poorly differentiated adenocarcinomas; in the latter histological type, the staining was either relatively weak or uneven as compared to that of the former, in which staining intensity was almost the same as that of tubular portions. In contrast to the cells forming tubules or clusters, sparsely infiltrating cells were generally stained faintly or hardly at all. There was no essential difference in intensity between cells infiltrated sparsely at different depths of the gastric wall.

*Immuno-electron microscopy staining of E-cadherin*

Expression of E-cadherin in gastric tissue specimens was examined in greater detail by immuno-electron microscopy (Figure 6). In normal gastric mucosa, the intercellular boundaries of adjacent epithelial cells were stained, but the apical borders with microvilli and the basal borders did not stain at all. In moderately differentiated adenocarcinomas, the cancer cell membranes stained deeply but unevenly and the free borders of cells also stained for E-cadherin. In poorly differentiated adenocarcinomas, the cell membrane stained only partially.

*E-cadherin expression of cancer cells in ascites or pleural effusion*

In order to investigate E-cadherin on free cancer cells of carcinomatous fluids from various adenocarcinomas (seven gastric adenocarcinomas, two pancreatic adenocarcinomas and two lung adenocarcinomas) were examined by immunofluorescence staining and phase-contrast microscopy (Table II). Except for one specimen of lung adenocarcinoma (patient No. 10), all others from carcinomatous fluids, even those

**Figure 8** Cell aggregation assay of cancer cells in ascites and pleural effusion. Cancer cells in ascites of gastric adenocarcinomas (patient Nos. 1,2) and pancreatic adenocarcinoma (patient No. 8) and those in pleural effusion of a lung adenocarcinoma (patient No. 10) hardly aggregated after 1 h incubation. In contrast, established gastric cancer cells, MKN 28, showed apparent aggregation. The degree of aggregation was represented by aggregation index $N_t/N_o$, where $N_o$ was the total particle numbers before incubation and $N_t$ was the total particle numbers after incubation for t min. O—O; patient No. 1, ●—●; patient No. 2, △—△; patient No. 8, ▲—▲; patient No. 10, □—□; MKN 28.

with positive immunoperoxidase staining of primary sites, were negative for E-cadherin by immunofluorescence staining. Cancer cells in all of those fluids, including those in pleural effusion from lung adenocarcinoma (patient No 10) generally showed no obvious cell to cell contact formation by phase-contrast microscopy. In Figure 7, some typical specimens selected from Table II, whose primary sites stained positively for E-cadherin, are shown in comparison to established gastric cancer cells. Staining for E-cadherin was not detected in any cancer cells of gastric adenocarcinomas and was detected throughout the cell membrane of lung adenocarcinoma, and none of these cancer cells showed apparent intercellular adhesions. To confirm that cells in carcinomatous fluids were interacting very loosely, cell aggregation assays were carried out. As shown in Figure 8, three specimens of ascites, two from gastric adenocarcinomas (patient No. 1,2) and one from pancreatic adenocarcinomas (patient No. 8) and specimens of pleural effusion which proved to be positive for E-cadherin (patient No. 10), exhibited no aggregation at all.

Crude membrane proteins prepared from cancer cells of the lung adenocarcinoma (patient No. 10) were then subjected to Western blot analysis with HECD-1. The expression of E-cadherin was clearly identified, although the mobility was slightly faster than intact E-cadherin of the gastric adenocarcinoma cell line MKN 28 (Figure 9).

**Figure 9** Western blot analysis of E-cadherin positive cancer cells from pleural effusion of a lung adenocarcinoma. Gastric adenocarcinoma cell line; MKN 28 **a**, which expressed functional E-cadherin showed a band at 124 kDa (arrow), whereas cancer cells from pleural effusion of lung adenocarcinoma (patient No. 10; **b**) exhibited a main band at 115 kDa (arrow) with several additional bands of lower molecular weight which probably represent degradated products. Molecular mass are in kilodalton.

### Discussion

E-cadherin expression and distribution in different histological types of gastric cancer was investigated by immunoperoxidase staining and immuno-electron microscopic staining, using an anti-E-cadherin monoclonal antibody (HECD-1). In normal gastric mucosa, the intercellular boundaries of epithelial cells except the zone of proliferation stained distinctly and evenly for E-cadherin. In well and moderately differentiated adenocarcinomas, staining intensity was almost the same as that of normal mucosa in both tubular portions and cell clusters regardless of the depth of infiltration, but was apparently low in singly infiltrating cells. The intensity of staining of poorly differentiated adenocarcinomas was less even in cell clusters, and still weaker in singly infiltrating cells. In signet ring cell carcinomas, where single-cell infiltration prevailed in all layers of the gastric wall, staining was hardly observed in any specimens. In general, in tumours of any histological type, almost all cells infiltrating singly were devoid of E-cadherins, although a very few sporadic cells stained. These results suggest that loss of expression of E-cadherin may be essential before cancer cells can dissociate from primary sites and infiltrate singly into the interstitial space.

In this context, freely infiltrating cells in carcinomatous fluids are more appropriate specimens. Immunofluorescence staining revealed that all cancer cells derived from malignant effusions associated with gastric adenocarcinomas, pancreatic adenocarcinomas and lung adenocarcinomas were also devoid of E-cadherin, although one primary lung adenocarcinoma stained positively for E-cadherin. Lack of E-cadherin expression in negative-staining cancer cells was further confirmed bv Western blot analysis. E-cadherin in positive-staining cells showed an apparent difference in mobility as compared to its intact counterpart, suggesting that this particular E-cadherin molecule may have been non-functional. In fact, no cell aggregation or intercellular compaction were observed in any of these carcinomatous fluid cells including the E-cadherin-positive lung adenocarcinoma cells.

Mechanisms for formation of caricnomatous fluids have not been clearly elucidated (Anisimov, 1982; Esaki *et al.*, 1990). In particular, there have been no extensive studies of cancer cells infiltrating to carcinomatous fluid. The results of the present investigation clearly indicate that lack of or dysfunction of E-cadherin was characteristic of cells in carcinomatous fluid. Furthermore, the fact that the primary sites were positive for E-cadherin in some cases with carcinomatous fluid, suggested the possibility that either they lost E-cadherin expression during detachment and infiltration process from the primary sites or that they represent some particular clones which lack E-cadherin at the primary sites and preferentially contribute to the malignant effusion.

Elucidation of the mechanisms for such depressed or impaired expression of E-cadherin in cancer cells should be an important task for understanding cancer progression. Our preliminary investigations conducted with signet ring cell lines revealed that in those with depressed E-cadherin expression on their cell surfaces, mRNA signals of E-cadherin were still detectable. These results suggest that the impairment of expression occurs at a post-transcriptional level. In order to establish the role of E-cadherin expression for detachment and infiltration of cancer cells, further studies on a wide variety of cancer tissues and animal models are warranted.

### References

ANISIMOV, V.M. (1982). Carcinogenesis and aging: III. The role of age in initiation and promotion of carcinogenesis. *Ext. Pathol.*, **22**, 131–147.

BEHRENS, J., MAREEL, M.M., VAN ROY, F.M. & BIRCHMEIER, W. (1989). Dissecting tumor cell invasion: epithelial cells acquire invasive properties after the loss of uvomorulin-mediated cell-cell adhesion. *J. Cell Biol.*, **108**, 2435–2447.

DUBAND, J.L., DUFOUR, S., HATTA, K., TAKEICHI, M., EDELMAN, G.M. & THIERY, J.P. (1987). Adhesion molecules during somitogenesis in the avian embryo. *J. Cell Biol.*, **104**, 1361–1374.

ESAKI, Y., HIRAYAMA, T. & HIROKAWA, K. (1990). A comparison of pattern of metastasis in gastric cancer by histologic type and age. *Cancer*, **65**, 2086–2090.

FRIXEN, U.H., BEHRENS, J., SACHS, M., EBERLE, G., VOSS, B., WARDA, A., LÖCHNER, D. & BIRCHMEIER, W. (1991). E-cadherin-mediated cell-cell adhesion prevents invasiveness of human carcinoma cells. *J. Cell Biol.*, **113**, 173–185.

HASHIMOTO, M., NIWA, O., NITTA, Y., TAKEICHI, M. & YOKORO, K. (1989). Unstable expression of E-cadherin adhesion moelcules in metastatic ovarian tumor cells. *Jpn. J. Cancer Res.*, **80**, 459–463.

HATTA, K., OKADA, T.S. & TAKEICHI, M. (1985). A monoclonal antibody disrupting calcium-dependent cell–cell adhesion of brain tissues: possible role of its target antigen in animal pattern formation. *Proc. Natl Acad. Sci. USA*, **82**, 2789–2793.

HATTA, K. & TAKEICHI, M. (1986). Expression of N-cadherin adhesion molecules associated with early morphogenetic events in chick development. *Nature*, **320**, 447–449.

JOHNSON, G.D. & NOGUEIRA ARAVJO, G.M.C. (1981). A simple method of reducing the fading of immunofluorescence during microscopy. *J. Immunol. Method*, **43**, 349–350.

LIOTTA, L.A. (1984). Tumor invasion and metastases: role of the basement membrane. *Am. J. Pathol.*, **117**, 339–348.

NOSE, A. & TAKEICHI, M. (1986). A novel cadherin cell adhesion molecule: its expression patterns associated with implantation and organogenesis of mouse embryos. *J. Cell Biol.*, **103**, 2649–2658.

SHIMOYAMA, Y., HIROHASHI, S., HIRANO, H., NOGUCHI, M., SHIMOSATO, Y., TAKEICHI, M. & ABE, O. (1989). Cadherin cell-adhesion molecules in human epithelial tissues and carcinomas. *Cancer Res.*, **49**, 2128–2133.

SHIRAYOSHI, Y., NOSE, A., IWASAKI, K. & TAKEICHI, M. (1986*a*). N-linked oligosaccharides are not involved in the function of a cell–cell binding glycoprotein E-cadherin. *Cell Struct. Funct.*, **11**, 245–252.

SHIRAYOSHI, Y., HATTA, K., HOSODA, M., TSUNASAWA, S., SAKIYAMA, F. & TAKEICHI, M. (1986*a*). Cadherin cell adhesion molecules with distinct binding specificities share a common structure. *EMBO J.*, **5**, 2485–2488.

TAKEICHI, M. (1977). Functional correlation between cell adhesive properties and some cell surface proteins. *J. Cell Biol.*, **75**, 464–474.

TAKEICHI, M., ATSUMI, T., YOSHIDA, C., UNO, K. & OKADA, T.S. (1981). Selective adhesion of embryonal carcinoma cells and differentiated cells by $Ca^{2+}$-dependent sites. *Dev. Biol.*, **87**, 340–350.

TAKEICHI, M., HATTA, K. & NAGAFUCHI, A. (1985). Selective cell adhesion mechanisms. In *Molecular Determinants of Animal Form*, Edelman, G.M. (ed.) pp. 223–233. Alan R. Liss: New York.

TAKEICHI, M., HATTA, K. & NAGAFUCHI, A. (1988). Identific ation of a gene family of cadherin cell adhesion molecules. In *Regulatory Mechanisms in Developmental Processes*, Eguchi, G., Okada, T.S. & Saxén, L. (eds) pp. 91–94. Elsevier Scientific Publishers Ltd: Ireland.

TOWBIN, H., STAEHELIN, T. & GORDON, J. (1979). Electrophoretic transfer of proteins from polyacrylamide gels to nitrocellulose sheets: procedure and some applications. *Proc. Natl Acad. Sci. USA*, **76**, 4350–4354.

YOSHIDA-NORO, C., SUZUKI, N. & TAKEICHI, M. (1984). Molecular nature of the calcium-dependent cell–cell adhesion system in mouse teratocarcinoma and embryonic cells studied with a monoclonal antibody. *Dev. Biol.*, **101**, 19–27.